# 2813. Impact of a Recent Change in Tobramycin Breakpoints (CLSI) on the Proportion of *Pseudomonas aeruginosa* Clinical Isolates that are Multidrug-Resistant: CANWARD, 2016 to 2021

**DOI:** 10.1093/ofid/ofad500.2424

**Published:** 2023-11-27

**Authors:** Andrew Walkty, Heather Adam, Melanie Baxter, Philippe Lagace-Wiens, James Karlowsky, George Zhanel

**Affiliations:** University of Manitoba, Winnipeg, Manitoba, Canada; University of Manitoba, Winnipeg, Manitoba, Canada; University of Manitoba, Winnipeg, Manitoba, Canada; University of Manitoba, Winnipeg, Manitoba, Canada; University of Manitoba, Winnipeg, Manitoba, Canada; University of Manitoba, Winnipeg, Manitoba, Canada

## Abstract

**Background:**

*Pseudomonas aeruginosa* (PA) is an important nosocomial pathogen. In 2023, tobramycin (TM) susceptibility breakpoints for PA were lowered (from ≤4 to ≤1 µg/mL) by the Clinical and Laboratory Standards Institute (CLSI). The purpose of this study was to evaluate whether this change will result in an increase in the proportion of PA clinical isolates that are considered multidrug-resistant (MDR).

**Methods:**

PA clinical isolates were obtained from patients evaluated at hospitals across Canada (January 2016 to December 2021) as part of an ongoing national surveillance study (CANWARD). Susceptibility testing was carried out using custom broth microdilution panels. MICs were interpreted using current (2023) CLSI breakpoints, with the exception of TM (interpreted using the 2022 and 2023 breakpoints). MDR PA were defined as isolates testing not susceptible at least three of the following: ceftazidime, ciprofloxacin, meropenem, piperacillin-tazobactam, and TM (assessed with both the 2022 and 2023 CLSI breakpoints).

**Results:**

1649 PA isolates were included in this study. The proportion of isolates testing susceptible to TM using the 2022 (S ≤4 µg/mL) and 2023 (S ≤1 µg/mL) CLSI breakpoints were 93.6% and 84.8%, respectively. Overall, 21.6% (356/1649) and 22.7% (374/1649) of PA isolates were MDR, using the 2022 and 2023 CLSI breakpoints. Below, the in vitro activities of common antipseudomonal antimicrobials versus PA isolates from this study (all and MDR subset) are presented.

**Conclusion:**

In this dataset, the proportion of MDR PA isolates was minimally altered by a recent change to the CLSI TM susceptibility breakpoints. A reduction in the proportion of PA isolates that were susceptible to TM using the new breakpoints was most pronounced for the MDR subset.

**Disclosures:**

**George Zhanel, PhD**, Avir: Grant/Research Support|Iterum: Grant/Research Support|Merck: Grant/Research Support|Orimed: Grant/Research Support|Paladin Labs: Grant/Research Support|Pfizer: Grant/Research Support|Verity: Grant/Research Support|Zambon: Grant/Research Support

**Table:** 

	PA Isolates (all) n = 1649			MDR PA isolates (2023 TM breakpoints) n = 374		
Antimicrobial	MIC_50_ (µg/mL)	MIC_90_ (µg/mL)	%S	MIC_50_ (µg/mL)	MIC_90_ (µg/mL)	%S
ceftazidime	4	>32	69.9	>32	>32	8.3
ciprofloxacin	0.25	4	71.9	2	16	25.7
meropenem	1	16	73.6	16	32	17.9
piperacillin-tazobactam	8	256	72.3	128	512	11.2
tobramycin (2022 breakpoints)	≤0.5	2	93.6	1	32	79.7
tobramycin (2023 breakpoints)	≤0.5	2	84.8	1	32	61.0
ceftazidime-avibactam	4	16	88.7	8	>16	56.4
ceftolozane-tazobactam	1	4	94.5	2	16	78.6
imipenem-relebactam	0.5	2	91.7	2	8	69.3

